# The Potential of Chitosan and Its Derivatives in Prevention and Treatment of Age-Related Diseases

**DOI:** 10.3390/md13042158

**Published:** 2015-04-13

**Authors:** Garry Kerch

**Affiliations:** Department of Materials Science and Applied Chemistry, Riga Technical University, Azenes 14/24, Riga, LV-1048, Latvia; E-Mail: garrykerch@inbox.lv; Tel.: +371-292-769-42

**Keywords:** chitosan, chitooligosacharides, age-related diseases, protein conformations

## Abstract

Age-related, diet-related and protein conformational diseases, such as atherosclerosis, diabetes mellitus, cancer, hypercholesterolemia, cardiovascular and neurodegenerative diseases are common in the elderly population. The potential of chitosan, chitooligosaccharides and their derivatives in prevention and treatment of age-related dysfunctions is reviewed and discussed in this paper. The influence of oxidative stress, low density lipoprotein oxidation, increase of tissue stiffness, protein conformational changes, aging-associated chronic inflammation and their pathobiological significance have been considered. The chitosan-based functional food also has been reviewed.

## 1. Introduction

According to the recent report of The Department of Economic and Social Affairs of the United Nations Secretariat [1] globally, the number of elderly (aged 60 years or over) is expected to increase from 841 million people in 2013 to more than 2 billion in 2050. The global share of older persons increased from 9.2 per cent in 1990 to 11.7 per cent in 2013 and proportion of the world population will reach 21.1 per cent by 2050. The share of older people aged 80 years or over (the “oldest old”) within the older population (aged 60 years or over) was 14 per cent in 2013 and is expected to reach 19 percent in 2050. There will be 392 million persons aged 80 years or over by 2050. Women live longer than men, so the older population will be mainly female.

The quality of life depends on the nutrition of the elderly population [2,3]. World Health Organization devoted special attention to nutrition for older people. Degenerative age-related diseases such as cardiovascular and cerebrovascular disease, diabetes, osteoporosis and cancer are the common diseases in older persons, and these diseases are also diet-affected [4]. Elevated serum cholesterol, a risk factor for cardiovascular diseases, is common in older people. It has been estimated that a 10% reduction in blood cholesterol concentration can reduce the risk of coronary heart disease by 30%. The decrease in salt and saturated fat intake can reduce blood pressure and blood cholesterol concentrations and can decrease the risk of cardiovascular disease. Increasing intake of fruit and vegetables by one to two servings daily could reduce cardiovascular risk by 30% [4].

Older people often suffer from impaired immunity [5,6,7]. Deficiency of trace elements zinc, iron, selenium, copper, Vitamins A, B, C, E have important impacts on immune responses. It has been reported [8] that chitooligosaccharide ascorbate is effective in compensation of deficiency in a number of minerals and vitamins. “The innate immune system is composed of a network of cells including neutrophils, NK and NKT cells, monocytes/macrophages, and dendritic cells that mediate the earliest interactions with pathogens. Age-associated defects are observed in the activation of all of these cell types, linked to compromised signal transduction pathways” [9]. Activation of intestinal T regulatory cells and homeostatic regulation of the gut microbiota may reduce low-grade inflammation in diet-related diseases [10] and, probably, also in age-related diseases.

**Figure 1:**
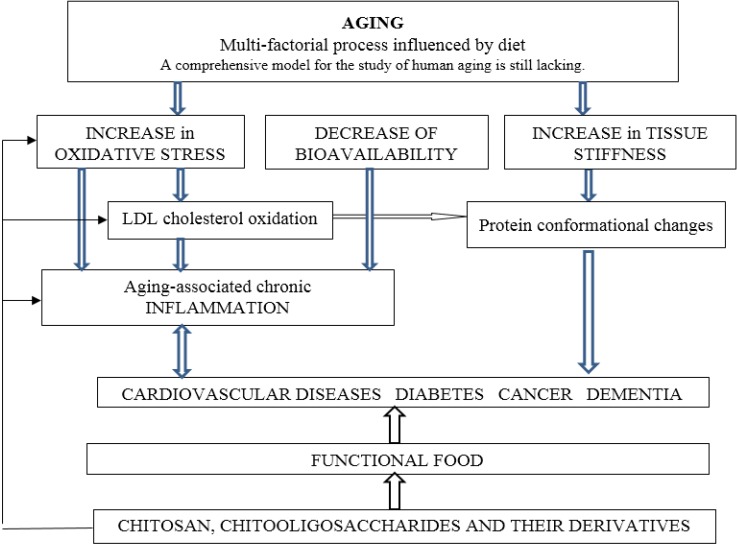
The potential effect of chitosan on age-related dysfunctions.

Functional foods and nutraceuticals with antioxidant, anti-inflammatory, anti-diabetic and anticancer properties may prevent age-related and diet-related diseases. Decline in immune response with aging and the role of nutrition in enhancing immunity have been reviewed recently [11]. Dietary components have the potential to improve immunity in ageing. The molecular mechanism underlying effects of diet and functional food on immunity remain to be determined [12]. The herbal polysaccharides with antioxidant and anti-inflammatory properties that are able to inhibit protein aggregation and to prevent associated age-related diseases have been reviewed recently [13] as well as marine derived polysaccharides [14]. Chitosan is a linear natural nontoxic cationic polysaccharide that due to its biocompatibility, biodegradability and cationic nature has advantages in biomedical applications over other neutral or negatively charged polysaccharides. The properties and various applications of chitosan and its derivatives and composites have been described in a number of recent books [15,16,17,18,19]. The potential of chitosan and its derivatives to prevent age-related diseases is presented in Figure 1.

The structure, properties and applications of chitosan, chitooligosaccharides (COS) and their derivatives have been described in many review papers [20,21,22,23,24,25,26,27,28,29,30]. Chitosan is bioactive cationic polysaccharide with antibacterial, antifungal, antioxidant, antidiabetic, anti-inflammatory, anticancer, and hypocholesterolemic properties. Chitosan is used in biomedical and food applications. This review paper focuses on the ability of chitosan and COS to prevent age-related dysfunctions.

## 2. Oxidative Stress

Antioxidant effects of chitin, chitosan and their derivatives have been reviewed recently and it was concluded that their antioxidant properties play a vital role in human health and nutrition [31]. The studies on antioxidant properties that have not been included in this recent review are discussed here.

Increased risk of oxidative stress in elderly people has been reported [32]. Increased levels of reactive oxygen species (ROS) can cause oxidative modifications of lipids, proteins, and DNA. Oxidative stress and inflammation are involved in the pathology of age-related diseases such as cardiovascular diseases, cancer, neurodegenerative diseases, rheumatoid arthritis, and diabetes mellitus (Figure 1). It is important to protect the cells from oxidative damage by ROS [33,34,35].

There is an urgent need to elucidate the role of oxidative stress in aging and to find promising perspectives on the efficacy of modulating agents for oxidative stress in treatment or prevention of age-related diseases. Perspectives on application of chitosan and its derivatives in the treatment of oxidative stress in age-related diseases have been considered in a number of research papers [31,36,37,38,39,40,41,42,43,44,45,46]. Researchers from Fukuyama University, Japan [43] concluded that chitosan has a direct antioxidant activity in systemic circulation by lowering the indices of oxidative stress in both *in vitro* and *in vivo* studies.

Researchers from Hubei University of Medicine, China, reported that COS inhibit ethanol-induced lipid peroxidation and glutathione depletion via the transcriptional activation of nuclear factor erythroid-2-related factor-2 (Nrf2) and reduction of the ethanol-induced phosphorylation of p38 MAPK, JNK and ERK [47]. Dietary chitosan supplementation attenuates isoprenaline-induced oxidative stress in rat myocardium [48] and the antiaging effect of dietary chitosan supplementation on glutathione-dependent antioxidant system in young and aged rats has been reported [49]. COS protect mice from oxidative stress [50]. Sulfated COS decrease intracellular ROS production. The researchers from Xi’an Jiaotong University, China [51] have reported protective effects of sulfated COS against hydrogen peroxide-induced damage in pancreatic β-cells MIN6. Sulfated COS significantly suppress nitric oxide (NO) production [52], the activity and mRNA expression of inducible NO synthase (iNOS), and the protein level of nuclear factor NF-κB protein p65 [52], which were activated by hydrogen peroxide H_2_O_2_. These results indicated the good anti-oxidative capacity of sulfated COS and the possible mechanism via the blockade of the nuclear factor NF-κB signaling pathway. The protective effects of sulfated COS against oxidative injuries in MIN6 cells depend both on their degree of substitution and concentration. The antioxidant action of low molecular weight chitosan was more effective in preventing the formation of carbonyl groups in plasma protein than high molecular weight chitosan [53]. The antioxidant activity of chitosan was increased after media milling. Rats fed media-milled chitosan showed increased superoxide dismutase activity [54]. Antioxidant activities of novel chitosan-caffeic acid, chitosan-ferulic acid, and chitosan-sinapic acid conjugates with different grafting ratios were investigated. The antioxidant activities of the conjugates were increased compared to the unmodified chitosan [55]. Researchers of Korea University, Seoul [56] reported that antioxidant property of chitosan green tea polyphenols complex induces transglutaminase activation in wound healing. Caffeic and ferulic acids were grafted onto chitosan by a free radical mediated method [57]. The novel compounds had improved peroxidation inhibition effects and increased free radical scavenging. The antioxidant activity of the phenol acids grafted N,O-carboxymethyl chitosan increased in order of chitosan ˂ N,O-carboxymethyl chitosan ˂ ferulic acid—N,O-carboxymethyl chitosan ˂ caffeic acid—N,O-carboxymethyl chitosan ˂ gallic acid—N,O-carboxymethyl chitosan [58]. COS reduce oxidative damage of DNA by inhibiting hydrogen peroxide H_2_O_2_ and AAPH radicals [59,60,61,62,63] and block degradation of inhibitory kappa B alpha (IκB-α) protein and translocation of nuclear factor kappa B (NF-κB) [63]. NF-κB translocates to the nucleus from cytoplasm when activated by stress, bacteria, inflammatory stimuli, cytokines, free radicals, carcinogens, and other agents. NF-κB regulates the expression of enzymes (such as COX-2 and iNOS), cytokines (such as TNF, IL-1, IL-6, IL-8), adhesion molecules and has been linked with age-related diseases such as atherosclerosis, diabetes, osteoporosis, Alzheimer’s disease, and cancer [64]. COS suppress NF-κB activation and so they are promising agents in prevention and treatment of age-related diseases.

The recent publications also show that grafting of natural antioxidant polyphenols on chitosan and COS derivatives, such as antioxidant sulfated COS [31], can result in the design of novel effective antioxidant nutraceuticals.

## 3. Inflammation

Oxidative stress and inflammation are involved in the pathology of age-related diseases such as cardiovascular diseases, cancer, neurodegenerative diseases, rheumatoid arthritis, and diabetes [33,34,35]. Chronic inflammation can be considered as a major risk factor for age-related diseases [65]. Oxidative stress results in upregulation of proinflammatory mediators (TNF-α, IL-1β, IL-6, COX-2, iNOS). Plasma TNF-α concentration is associated with aging and the risk of diabetes mellitus [66].

COS inhibit the expression of IL-6 in lipopolysaccharide (LPS)-induced human umbilical vein endothelial cells (HUVECs). The pre-treatment of HUVECs with COS inhibited the LPS-induced over-expression of phosphorylated p38 mitogen-activated protein kinase (MAPK), phosphorylated ERK1/2 and nuclear factor NF-κB. COS prevented degradation of inhibitory protein IκBα in nuclear factor NF-κB and translocation of NF-κB from cytoplasm to nucleus [67].

COS inhibit LPS-induced over-expression of inflammatory cytokines IL-6 and TNF-α also in RAW264.7 macrophage cells through blockade of MAPK and PI3K/Akt signaling pathways and suppress the activation of NF-κB and activator protein-1 (AP-1) [68]. The similar behavior has been reported for sulfated COS [52]. It has been suggested recently [69] that COS block LPS-induced *O*-GlcNAcylation (a dynamic modification of proteins by β-linked *N*-acetylglucosamine) of NF-κB and endothelial inflammatory response.

Adhesion molecules are involved in the adhesive interaction between endothelial cells and monocytes in inflammation. COS down regulate the expression of adhesion molecules E-selectin and ICAM-1 by inhibiting the phosphorylation of MAPKs and the activation of NF-κB in LPS-treated porcine iliac artery endothelial cells [70]. Sulfated chitosan inhibits P-selectin-mediated HL-60 leukocyte adhesion. Sulfochitosans exhibit inhibitory activity in the order: heparin > *N*-sulfated/6-*O*-sulfated chitosan ≥ 3-*O*,6-*O*-sulfated chitosan > 6-*O*-sulfated chitosan >> *N*-sulfated chitosan. So, it can be concluded that the sulfation of the double site in chitosan is essential for efficient inhibition of P-selectin-mediated HL-60 leukocyte adhesion [71].

The effects of chitosan and quaternized chitosan on production of IL-1β and TNF-α in LPS-stimulated human periodontal ligament cells has been studied [72]. Chitosan inhibited the production of IL-1β and TNF-α and quaternized chitosan increased IL-1β and TNF-α production.

COS attenuate ocular inflammation in rats with experimental autoimmune anterior uveitis [73] and prevented retinal ischemia and reperfusion injury via reduced oxidative stress and inflammation [74].

The elevated plasma glucose, TNF-α, and IL-6 in diabetic rats were decreased after 10 weeks of chitosan feeding [75]. Biomarkers provide insights into complex disease mechanisms and can help to develop novel nutraceuticals. Evidently, it does not mean that it is enough to decrease the content of inflammatory biomarkers to completely prevent and treat diabetes and other age-related diseases.

COS inhibited the release and expression levels of inflammatory cytokines TNF-α, IL-6 and IL-1β in LPS-stimulated BV2 microglia. COS also attenuated the production of NO and prostaglandin E2 (PGE2) by inhibiting iNOS and cyclooxygenase-2 (COX-2) expressions [76]. It has been confirmed recently that COS decrease the levels of NO, TNF-α and IL-1β, released from LPS-stimulated RAW264.7 cells by inhibiting the activation of the NF-κB pathway [77].

Chitosan decreased serum TNF-α and leptin levels in high fat fed rats [78]. NF-κB activation, and levels of TNF-α and IL-6 in colonic tissues, were suppressed in mice with inflammatory bowel disease receiving COS [79]. It has been reported that the oral intake of COS by elderly volunteers decreased inflammatory cytokines TNF-α and IL-1β levels [80]. COS added to diet have been reported as calcium fortifiers [81] in ovariectomised rats and this effect has been related to COS capacity to down-regulate mRNA and protein expression of COX-2, a key mediator linking inflammation and osteoporosis. It has been demonstrated *in vivo* that COS are able to induce an anti-inflammatory effect mediated by cyclooxygenase inhibition and reduction of prostaglandins [82].

The anti-inflammatory and anticancer properties of chitin oligosaccharide and chitosan oligosaccharide have been recently reviewed [83].

## 4. Diabetes Mellitus

Antidiabetic effects of chitin, chitosan and their derivatives have been reviewed recently [84]. It has been concluded that chitosan and its derivatives have the potential to be used in several antidiabetic therapeutic applications and future research should be directed to enhance the effectiveness of novel chitosan derivatives and chitosan-based compounds to be used as potent nutraceuticals for prevention of diabetes and diabetes-related complications. So, the studies on antidiabetic properties of chitosan, COS and their derivatives that have not been included in this recent review are discussed here with special attention given to application in age-related diabetes.

Protein-rich diet is recommended at present for the treatment of elderly malnutrition. However, it was demonstrated recently in a large population-based prospective study by scientists of Lund University [85] that high intakes of protein and processed meat are associated with increased incidence of type 2 diabetes. At the same time, the intake of fiber-rich bread and cereals was inversely associated with type 2 diabetes. Insulin independent diabetes mellitus, the type II diabetes, is a serious global problem getting worse every year. The team of researchers from University of Southern California led by Longo [86] also reported that high protein intake was associated with reduced cancer and overall mortality in respondents over 65, but a five-fold increase in diabetes mortality across all ages. It must be also taken into account that diabetes mellitus is a risk factor for incidence of age-related dementia, Alzheimer’s disease, and cardiovascular diseases. So, a novel, complex balanced, more safe protein-rich diet and functional food to tackle malnutrition in elderly with lower risk of diabetes development must be designed.

The use of antioxidants reduces oxidative stress and alleviates diabetic complications [87]. Oxidative stress common for older people can lead to increased lipid peroxidation and development of diabetes mellitus [88,89]. TNF-α expression in the insulin resistant subjects and the diabetic patients was four-fold higher than in the insulin sensitive subjects [90]. Plasma TNF-α concentration is significantly associated with advancing age and it predicts the impairment in insulin action with advancing age [66]. Miura and coworkers [91] reported that chitosan had blood glucose-lowering and lipid-lowering effects in neonatal streptozotocin-induced diabetic mice. Hayashi and Ito [92] reported that low molecular-weight chitosan lactate had an antidiabetic effect also in obese diabetic KK-Ay mice. Chitosan may possess a potential for alleviating type-1 diabetic hyperglycemia through the decrease in liver gluconeogenesis and increase in skeletal muscle glucose uptake and use [93]. A randomized, double-blind, placebo-controlled clinical trial on 12 week supplementation of COS in subjects with prediabetes showed a significant decrease in the serum glucose level [94]. The effects of chitosan-oligosaccharide (GO2KA1) on postprandial blood glucose levels in adults with normal blood glucose levels have been recently reported [95,96]. GO2KA1 reduced postprandial blood glucose level due to the decrease of absorption of glucose in the small intestine as a result of carbohydrate hydrolyzing enzyme inhibition. Hsieh and coworkers [75] demonstrated that chitosan reduces plasma adipocytokines and lipid accumulation in liver and adipose tissues and ameliorates insulin resistance in diabetic rats. After 10 weeks of feeding, the elevated plasma glucose, TNF-α, and IL-6 and lower adiponectin levels caused by diabetes were effectively reversed by chitosan treatment. Chitosan feeding also reduced hepatic triglyceride and cholesterol contents. Hsieh and coworkers consider that long-term administration of chitosan may reduce insulin resistance through suppression of lipid accumulation in liver and adipose tissues and amelioration of chronic inflammation in diabetic rats.

## 5. Hypercholesterolemia

Low-density lipoprotein (LDL) oxidation is associated with coronary atherosclerosis. High levels of cholesterol oxidation products in oxidized LDL are toxic for endothelial cells [97,98]. Removal of oxidized LDL is an important part of the protective role of the macrophage in the inflammatory response [99]. Mediators of inflammation such as TNF-α, IL-1, and macrophage colony-stimulating factor increase binding of LDL to endothelium and smooth muscle. Antioxidants have an anti-inflammatory effect by preventing the up-regulation of adhesion molecules for monocytes. Antioxidants increase the resistance of human LDL to oxidation. High-density lipoproteins inhibit cytokine-induced expression of endothelial cell adhesion molecules [100].

Hypocholesterolemic effects of chitosan have been reported in many publications [101,102,103,104,105,106,107,108,109,110,111]. Recently, it has been demonstrated that the effect of media-milled chitosan on the decrease of serum triacylglycerol, total cholesterol and LDL cholesterol is higher compared to chitosan [54]. It has been also demonstrated recently that total cholesterol content in mice blood fed during 12 weeks with γ-irradiated chitosan (30–100 kGy) was significantly lower than that of the control [112]. The role of chitosan in lipid lowering treatment has been discussed recently by Patti and coworkers [113].

## 6. Cancer

In a recent review [31], it has been concluded that formation of cancer cells can be induced by free radicals. Hence, antioxidant properties of chitosan and its derivatives can be used to reduce the chance for the formation of cancer in the human body.

The anticancer properties of chitin and chitosan oligosaccharides have been recently reviewed [83]. Chitin and chitosan oligosaccharides have been evaluated as functional foods against cancer. The studies on anticancer properties that have not been included in this recent review are discussed here.

Polyphenols, such as curcumin or resveratrol, are effective natural antioxidants and their bioavailability can be essentially improved by encapsulation in chitosan-based nanoparticles [114] to be delivered to cancer cells. It has been also reported that novel cationic curcumin-chitosan poly (butyl cyanoacrylate) nanoparticles synthesized by emulsion polymerization, can improve the bioavailability of hydrophobic drug curcumin, suppress hepatocellular carcinoma growth and inhibit tumor angiogenesis efficiently *in vitro* and *in vivo* [115]. Rejinold and coworkers [116] fabricated curcumin with biodegradable thermoresponsive chitosan-g-poly (*N*-vinylcaprolactam) nanoparticles (TRC-NPs) for cancer drug delivery. Their results indicate that novel curcumin-loaded TRC-NPs could be a promising candidate for cancer drug delivery. The anticancer effect can be explained by activation of apoptosis signaling (curcumin inhibits Bcl2 and activates caspase 9 to induce apoptosis) and blockade of cell proliferation signaling pathways (such as MAP kinase pathway, AKT pathway and mTOR pathways) [117,118,119,120,121]. Researchers from Universidade Federal de Santa Catarina, Florianópolis, Brazil and Université Grenoble Alpes, Grenoble, France published a number of papers describing curcumin-loaded chitosan-coated nanoparticles. Curcumin-loaded chitosan-coated nanoparticles can be used for the local treatment of oral cavity cancer [122]. The mucoadhesive properties of chitosan due to its polycation nature have been used to prepare films containing chitosan-coated nanoparticles for buccal delivery of curcumin [123]. Xyloglucan-block-poly (ϵ-Caprolactone) copolymer nanoparticles coated with chitosan were developed as biocompatible mucoadhesive drug delivery system [124]. Chitosan interacts with mucin through electrostatic forces between protonated amino groups of chitosan and negatively charged groups of mucin [125].

It has been also reported that oral administration of chitosan based nanoformulated green tea polyphenol EGCG effectively inhibits prostate cancer cell growth [126]. EGCG has been encapsulated also into chitosan-coated nanoliposomes and anticancer effects are expected in the treatment of breast cancer [127].

The curcumin/5-fluorouracil loaded thiolated chitosan nanoparticles showed enhanced anticancer effects on colon cancer cells *in vitro* and improved the bioavailability of the drugs *in vivo* [128]. The 5-fluorouracil and curcumin released from the N,O-carboxymethyl chitosan nanoparticles also produced enhanced anticancer effects *in vitro* in colon cancer cells HT 29 and improved plasma concentrations under *in vivo* conditions in mouse model [129].

**Figure 2:**
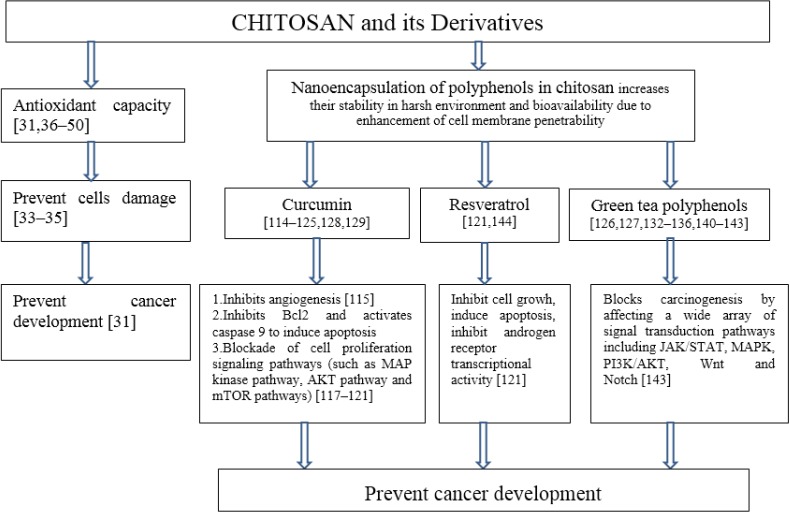
The possible anticancer effect of chitosan and polyphenols encapsulated in chitosan nanoparticles.

Chitosan-based nanoparticles for tumor-targeted drug delivery that display a range of useful properties such as biocompatibility, biodegradability, excellent cell membrane penetrability, high drug-carrying capacities, pH-dependent unloading and prolonged circulating time have been recently reviewed by Prabaharan [130]. The possible anticancer effect of chitosan and polyphenols encapsulated in chitosan nanoparticles is presented in Figure 2.

## 7. Nanomedicine

Application of chitosan as drug carriers has been reviewed [130,131]. Decrease of bioavailability of nutrients with aging is an important problem leading to age-related dysfunctions. So, it is a great challenge to use chitosan as carriers of nutraceuticals that are able to delay or prevent age-related dysfunctions.

Catechins found in green tea have demonstrated antioxidant, cardioprotective, neuroprotective and anticancer effects. However, the oral administration of these oxidation-sensitive compounds is limited by the harsh environment of the gastrointestinal tract, their poor stability and intestinal absorption. Encapsulation in chitosan nanoparticles enhance the intestinal absorption of the green tea catechins (+)-catechin and (−)-epigallocatechin gallate (EGCG) [132]. Researchers from Monash University, Australia, considered that the mechanism by which absorption was enhanced was not through an effect of chitosan nanoparticles on intestinal paracellular or passive transcellular transport or an effect on efflux proteins but was likely due to stabilization of catechins after encapsulation. Oral absorption of encapsulated EGCG has been evaluated in Swiss Outbred mice. Administration of the chitosan nanoparticles enhanced the plasma exposure of total EGCG by a factor of 1.5 relative to an EGCG solution [133]. Nanochemoprevention by encapsulation of (−)-epigallocatechin-3-gallate with bioactive peptides/chitosan nanoparticles for enhancement of its bioavailability also has been reported [134].

Tang and coworkers [135] consider that chitosan nanoparticles with a positive surface charge could transiently open the tight junctions between Caco-2 cells and thus increase the paracellular transport of tea catechins. They prepared nanoparticles composed of chitosan and an edible polypeptide, poly (γ-glutamic acid) (γ-PGA) for the delivery of tea catechins and demonstrated that chitosan/γ-PGA nanoparticles can be effective as a carrier for oral delivery of tea catechins with effective antioxidant activity.

It must be taken into account [136,137] that in comparison to the free-soluble polymers, the nanoparticles prepared by ionic gelation of the chitosan and its quaternized derivatives can have much lower effect on decreasing the transepithelial electrical resistance by opening of the tight junctions and on the permeability of cell layers in a Caco-2 cell system due to the reduced available amount of positive charge at the surface of the nanoparticles. However, no differences in cell permeability were detected between chitosan solution and chitosan nanoparticles on Calu-3 cells [138].

Chitosan coating prevents the aggregation of bovine serum albumin (BSA)—epigallocatechin gallate (EGCG) nanoparticles at pH 4.5–5.0 and may improve the absorption of EGCG [139]. Chitosan coating has been used also for (−)-epigallocatechin-3-gallate (EGCG) encapsulated nanostructured lipid carriers [140,141]. EGCG has been encapsulated into chitosan-coated nanoliposomes and a potential breakthrough in the prevention or even treatment of breast cancer has been expected [127]. Folate conjugated chitosan coated EGCG nanoparticles were prepared using the ionic gelation method with folic acid modified carboxymethyl chitosan and chitosan hydrochloride as carriers of catechin EGCG [142]. EGCG blocks carcinogenesis by affecting a wide array of signal transduction pathways including JAK/STAT, MAPK, PI3K/AKT, Wnt and Notch [143].

Chitosan/poly (d,l-lactic-co-glycolic acid) (PLGA) microcapsules were prepared by W/O/W double emulsion method and nutraceutical resveratrol was encapsulated into microcapsules [144].

## 8. Neurodegenerative Diseases

Cases of dementia and Alzheimer’s are expected to almost double every 20 years to around 66 million in 2030 and over 115 million in 2050 [145].

A chronic inflammatory response associated with Aβ and IL-1β is responsible for the pathology of Alzheimer’s disease. The polyphenol EGCG binds directly to a large number of proteins that are involved in protein misfolding diseases and inhibits their fibrillization [146]. Water-soluble chitosan inhibits the production of pro-inflammatory cytokine in human astrocytoma cells activated by Aβ and IL-1β and may reduce and delay the pathological events associated with Alzheimer’s disease [147].

**Figure 3:**
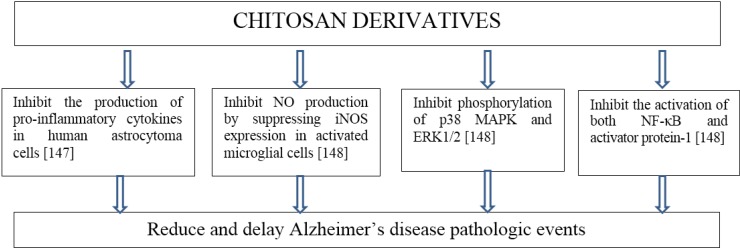
The possible effect of chitosan oligosaccharide on Alzheimer disease pathology.

The effect of COS on NO production in LPS induced N9 microglial cells has been studied [148]. Pretreatment with COS could inhibit NO production by suppressing iNOS expression in activated microglial cells. COS inhibited LPS-induced phosphorylation of p38 MAPK and ERK1/2. COS pretreatment could also inhibit the activation of both NF-κB and activator protein-1 (AP-1). The possible effect of chitosan oligosaccharide on Alzheimer disease pathology is presented in Figure 3.

## 9. Protein Conformational Diseases

The changes in protein conformations lead to protein conformational diseases that are also age-related diseases—diabetes mellitus, cataract, Alzheimer’s disease, dementia, and atherosclerosis. Protein oxidation or glycation induces protein unfolding, adhesion of unfolded proteins to the arterial wall with increased arterial stiffness and the initiation of vascular inflammation and atherosclerosis [149,150,151,152,153,154]. Atherosclerotic plaques contain oxidized LDL which has amyloid properties [155] and activates platelets [151]. Misfolded proteins support platelet activation and aggregation leading to protein conformational diseases [156,157].

The presence of water at the protein-lipid interface of membrane proteins can affect the changes in protein conformations. Cholesterol is known to reduce the water content of lipid bilayers. Changes in the degree of hydration can lead to changes in protein conformation [158]. Chitosan prevents formation of carbonyl and hydroperoxide groups in human serum albumin exposed to peroxyl radicals and inhibits conformational changes in the protein, assessed by absorption spectrum and intrinsic fluorescence [159].

**Figure 4:**
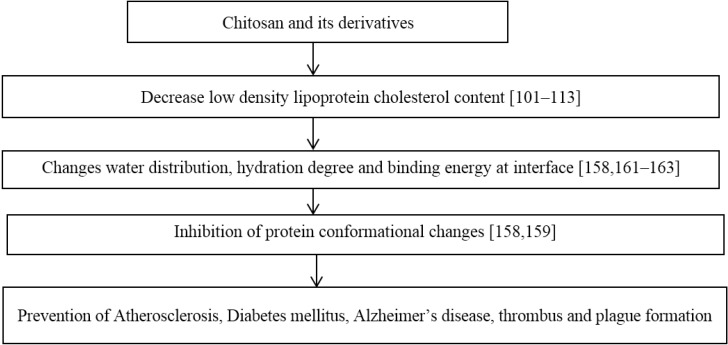
The possible influence of chitosan on low density lipoprotein cholesterol content, hydration, protein conformation, and protein conformational diseases.

Proteins can be denatured by various stresses. Various additives are known to minimize the damage and to enhance the stability of proteins [160]. So, the possible mechanism of beneficial effect of COS in protein conformational diseases can be also due to their ability to prevent conformational changes in proteins. Tissue dehydration with aging [161] can cause protein conformational changes. The possible effect of chitosan on protein conformational diseases is presented in Figure 4. The need for further research in this area is evident.

It has been demonstrated that the presence of chitosan can change water migration and distribution in complex food systems such as bread [162] and can change interaction and water distribution between gluten and starch. Also, it has been shown that chitosan can prevent platelet adhesion to implants if water molecules are tightly bound to chitosan macromolecules and do not prevent platelet adhesion if water molecules at interface are free or loosely bound to the chitosan coated surface [163]. In both cases, interaction with protein depends on the water binding energy to chitosan macromolecules.

## 10. Chitosan Containing Food

It would be desirable if health promoting additives are used in everyday food and the elderly will not be forced to make drastic dietary changes. So, bread and dairy food containing chitosan and its derivatives could be the most appropriate functional food.

### 10.1. Bread Containing Chitosan and Its Derivatives

The papers published before 2007 have been reviewed by No *et al.* [164] and they reported that chitosan [165,166] and chitosan oligosaccharide [167] coatings extend shelf life of bread. The additives of chitosan [168] and carboxymethyl chitosan [169] also have been reported to extend shelf life of bread. The extension of bread shelf life has been explained by inhibiting microbial growth and by retarding starch retrogradation. Chitosans with higher molecular weight (30 and 120 kDa) have been reported to be more effective than chitosans with lower molecular weight (1 and 5 kDa) in extending the shelf life of bread.

The patients receiving chitosan-containing bread during 12 weeks decreased their mean levels of LDL-cholesterol and significantly increased their mean levels of HDL-cholesterol at the end of the study [101].

However, it has been also reported that chitosan increases the rate of bread staling [162]. Chitosan oligosaccharides and low molecular weight chitosan increase bread crumb staling rate to a much lesser extent than does middle molecular weight chitosan [170].

The addition of microcrystalline chitin increased specific loaf volume of white bread and protein fortified breads [171]. The properties of bread containing chitosan have been studied in a number of publications [172,173,174,175,176,177].

Chitosan has been approved as a food additive in Korea and Japan since 1995 and 1983, respectively. US FDA approved for chitosan GRAS status [154], so chitosan also can be used as a food additive.

### 10.2. Dairy Products Containing Chitosan and Its Derivatives

The use of chitosan in dairy products has been reported in a number of publications [164,178,179,180,181,182,183,184,185,186,187,188,189,190]. Microencapsulation with chitosan coating enhanced the survival of probiotic bacteria significantly in ice cream during storage compared to free cells [181]. Inhibitory effect of chitooligosaccharide on fermentation of sour cream [184] and inhibitory effect of chitosan on post-acidification of set yoghurt during cold storage [185] have been reported. Viscosity of sour cream increases with the increase of concentration of high molecular weight chitosan and anomalous viscosity decrease was observed with the increase in concentration of chitooligosaccharide [184].

## 11. Conclusions and Perspectives

Chitosan and COS due to their antioxidant, anti-inflammatory, antidiabetic, and anticancer properties show promising potential to be used in prevention, delay, mitigation and treatment of age-related dysfunctions and diseases. Hypocholesterolemic properties of chitosan decrease the risk of atherosclerosis and other cardiovascular dysfunctions common in elderly population. Chitosan ability to decrease serum total-cholesterol and LDL cholesterol levels, as well as to prevent their oxidation, changes water molecules distribution at biointerfaces and influences the conformation of proteins. So, chitosan has the potential to prevent protein conformational diseases that are also related with advanced aging. Mucoadhesive properties of chitosan can be applied in nanomedicine with potential to improve effectiveness of nutraceuticals and drug delivery systems. Combination of chitosan and COS with natural antioxidant polyphenols is promising. COS suppress nuclear factor NF-κB activation and translocation of NF-κB from cytoplasm to nucleus that has been linked with a number of age-related diseases.

Development of novel COS derivatives such as sulfated, carboxylated and phenolic acid conjugated COS and their application in novel nanoparticulated systems, functional foods, and nutraceuticals can essentially increase bioavailability and stability of bioactive components. Mucoadhesive films containing chitosan-coated nanoparticles can find novel applications in nanomedicine. Breakthrough results in delay and prevention of age-related dysfunctions can be expected in the future.
